# Structural Progression in Patients with Definite and Non-Definite Arrhythmogenic Right Ventricular Cardiomyopathy and Risk of Major Adverse Cardiac Events

**DOI:** 10.3390/biomedicines12020328

**Published:** 2024-01-31

**Authors:** Areej Aljehani, Shanat Baig, Tania Kew, Manish Kalla, Laura C. Sommerfeld, Vaishnavi Ameya Murukutla, Larissa Fabritz, Richard P. Steeds

**Affiliations:** 1 Institute of Cardiovascular Sciences, University of Birmingham, Birmingham B15 2TT, UK; 2 Department of Cardiology, University Hospitals Birmingham NHS Foundation Trust, Queen Elizabeth, Birmingham B15 2GW, UK; 3 Echocardiography Cardiovascular Technology (ECVT) Program, King Saud bin Abdulaziz University for Health Sciences, Riyadh 11481, Saudi Arabia; 4 University Center of Cardiovascular Science & Department of Cardiology, University Heart and Vascular Center and University Medical Center Hamburg-Eppendorf, 20246 Hamburg, Germany; 5 German Centre for Cardiovascular Research DZHK, Partner Site Hamburg/Kiel/Luebeck, 20246 Hamburg, Germany

**Keywords:** arrhythmogenic right ventricular cardiomyopathy, major adverse cardiac events, single-centre observational cohort study, structural progression, echocardiography, disease progression, sudden cardiac death, male sex

## Abstract

Arrhythmogenic right ventricular cardiomyopathy (ARVC) is a rare inherited disease characterised by early arrhythmias and structural changes. Still, there are limited echocardiography data on its structural progression. We studied structural progression and its impact on the occurrence of major adverse cardiovascular events (MACE). In this single-centre observational cohort study, structural progression was defined as the development of new major or minor imaging 2010 Task Force Criteria during follow-up. Of 101 patients, a definite diagnosis of ARVC was made in 51 patients, while non-definite ‘early’ disease was diagnosed in 50 patients. During 4 years of follow-up (IQR: 2–6), 23 (45%) patients with a definite diagnosis developed structural progression while only 1 patient in the non-definite (early) group gained minor imaging Task Force Criteria. Male gender was strongly associated with structural progression (62% of males progressed structurally, while 88% of females remained stable). Patients with structural progression were at higher risk of MACE (64% of patients with MACE had structural progression). Therefore, the rate of structural progression is an essential factor to be considered in ARVC studies.

## 1. Introduction

Arrhythmogenic right ventricular cardiomyopathy (ARVC) is an inherited heart muscle disease, which is characterised by progressive myocyte loss and replacement by fibrous tissue, primarily of the right ventricular (RV) myocardium [1], but often also involving the left ventricle (LV) as well. ARVC is associated with an increased risk of ventricular arrhythmias (VA), and sudden cardiac death (SCD), particularly in young, otherwise healthy athletes [2]. In advanced disease, ARVC results in RV and LV dilatation and dysfunction [3]. Although genetic testing can identify individuals at risk of developing ARVC [4], predicting the risk of major adverse cardiac events (MACE) remains a challenge. Clinical and histological studies have shown that ARVC is a progressive disease, as no structural heart defects are detectable at birth [5]. So-called ‘hot phases’ have been observed in some patients, during which patients with ARVC may become symptomatic, associated with the release of circulating biomarkers including high-sensitivity troponin, and subsequent changes on imaging [6]. Accurate evaluation of the progression of ARVC by echocardiographic assessment of ventricular size and function, as recommended by the 2023 ESC Guidelines for the Management of Cardiomyopathies [7], may help to improve the prediction of major adverse cardiac events and guide future therapy. The aim of this longitudinal study was to assess structural progression in different stages of ARVC with a focus on transthoracic echocardiography to determine the association between structural progression and MACE and to identify markers of disease progression.

## 2. Methods

### 2.1. Study Population

The study population was recruited retrospectively from a single centre: the Inherited Cardiac Conditions clinic at the University Hospitals Birmingham, United Kingdom. Patients were classified as having either definite ARVC or non-definite ‘early’ disease, as per the modified Task Force Criteria for Diagnosis from 2010 [8]. We included individuals aged 18 years and older who had undergone at least two 12-lead electrocardiographic (ECG) and transthoracic echocardiographic examinations, with an interval of at least 6 months between each examination. The baseline examination was the first available echocardiogram, and the last follow-up echocardiogram was defined as the most recent evaluation before December 2022, or the final before cardiac transplantation or death. Patients with non-definite ARVC who did not undergo genetic testing were excluded from this analysis. We analysed all available echocardiographic examinations between the baseline and last follow-up. We extracted data on demographic and clinical characteristics and disease outcomes from the clinical data system. We categorised patients into those with a history of competitive and non-competitive sports based on the criteria established in reference [9].

### 2.2. Definition of Outcomes

MACE was defined as one of the following: ventricular fibrillation (VF), sustained ventricular tachycardia (Sus VT), appropriate implantable cardio-defibrillator (ICD) therapy (shock/anti-tachycardia pacing (ATP)), heart failure (defined as decompensated heart failure, cardiac index by heart catheter, HF medication and symptoms), cardiac transplantation, or death.

### 2.3. Structural Evaluation

Transthoracic echocardiography was performed by experienced echocardiographers accredited by the British Society of Echocardiography (BSE). All 306 echocardiograms between baseline and last follow-up were re-analysed by a single observer blinded to the clinical characteristics of the patients and the initial diagnosis, with standard measurements made in accordance with the most recent requirements of the published BSE guidelines [10] using IntelliSpace Cardiovascular technology (ISCV; Philips, Amsterdam, The Netherlands). Measurements were taken for the right ventricular (RV) outflow tract (RVOT) diameter in the parasternal long (PLAX) and short-axis (PSAX) views, as well as for the RV basal and mid-cavity size and RV fractional area change (RVFAC) in the modified apical four-chamber view. Additionally, LV ejection fraction (LVEF) was assessed using the biplane Simpson method.

Cardiovascular magnetic resonance (CMR) imaging was performed at baseline in 64 patients using a 1.5T Avanto scanner (Siemens Healthcare, Erlangen, Germany) following standardised protocols [11]. Right ventricular ejection fraction (RVEF) and the left ventricular ejection fraction (LVEF) along with right ventricular volumes (RVEDV) and left ventricular volumes (LVEDV) during end-diastole and end-systole analysis were performed using cvi42^®^ (version 5.3.6, Circle Cardiovascular Imaging, Calgary, AB, Canada) by a single independent operator (AA) who was blinded to clinical and diagnostic characteristics. For ventricular volume analysis, the endocardial border was defined as the largest and smallest cavity volumes, at end diastole and end systole. Late gadolinium enhancement (LGE) imaging was performed 7–10 min after administering 0.15 mmol/kg of gadolinium-based contrast agent (Gadovist Bayer Healthcare). Only 12 patients underwent a follow-up scan.

The presence of major and minor imaging criteria was assessed according to the 2010 Modified Task Force Criteria [8]. Patients were classified as definite and non-definite according to these criteria, with non-definite including both borderline and possible cases. Patients were also classified into those with progressive structural disease or stable disease. Structural progression was defined as the development of new major/minor TFC imaging diagnostic criteria on echocardiography or CMR during follow-up.

### 2.4. ECG Measurements

Patients underwent 12-lead ECG and signal-averaged ECG (SAECG) at the ICC clinic using a General Electric MAC 5500 HD Resting ECG System (Marquette, Milwaukee, WI, USA) with high-pass filter of (40–250 HZ). These were assessed for the heart rate, duration of standard ECG intervals PR, QRS and QT interval presence or absence of repolarisation and depolarisation criteria. Late potential criteria were assessed as defined by 2010 TFC [8]. 24-h Holter ECG monitoring was performed in a subgroup to detect premature ventricular contraction (PVC) and was considered abnormal if showing >500 premature ventricular contractions (PVC)/24 h.

Electrical progression was defined as development new minor/major depolarisation or repolarisation criteria or >500 PVC/24 h during following up.

### 2.5. Risk Estimation

We applied an ARVC risk calculator scoring system as developed in a multinational cohort in 2019 for prediction of SCD and sustained ventricular arrhythmias in stable and progressive structural ARVC patients [12]. It provides a personalised assessment for patients without history of sustained ventricular arrhythmias and it has been validated on external cohorts and successfully predicted sustained ventricular arrhythmias [13]. The scoring system includes male gender, history of syncope, number of T-wave inversions, number of PVC in 24 h Holter ECG, non-sustained VT, and right ventricular ejection fraction by CMR. For this retrospective purpose, we applied the ARVC risk calculator on our cohort for stable and progressive structural ARVC patients for correlation with the experience of MACE during follow-up in this cohort.

### 2.6. Patient Cohort

A total of 168 patients with either ARVC or at risk of developing ARVC as the main diagnosis attended the ICC clinic between 2010– start of 2022. Out of these, 67 patients were excluded from this study for the following reasons: lack of genetic test results for patients with non-definite ARVC (n = 32); lack of follow-up echocardiography at the hospital (n = 14: of those, 6 did not attend, 5 had been transferred elsewhere, 2 were discharged aged over 65 years without disease, and 1 had died); paired data less than 6 months apart (n = 11); and patients scheduled to have follow-up after 2022 (n = 10). A total of 101 patients with at least one paired ECG and echocardiogram were included in the study. A definite diagnosis of ARVC was made in 51 patients, while non-definite ‘early’ disease was diagnosed in 50 patients. This was a snapshot analysis. Follow-up was analysed until December 2022.

### 2.7. Statistical Analysis

For comparisons between groups, normally distributed continuous variables were analysed using independent-samples t-tests and presented as mean ± standard deviation (SD). Non-normally distributed variables were analysed using Mann–Whitney U tests and summarised as medians and interquartile ranges (IQRs), with categorical variables analysed using Fisher’s exact or Chi-squared tests for variables with two or more than two levels, respectively, and summarised as frequencies and percentages of the total population. Differentiation between normally and non-normally distributed data was assessed via the Shapiro–Wilk test.

For analysis of serial measurements, changes in categorical variables between baseline and the most recent follow-up were assessed using McNemar’s test. For continuous variables, longitudinal trends were analysed using a generalised estimating equation approach to account for the non-independence of repeated assessments on the same patient. These models assumed an AR (1) correlation structure, to account for within-patient correlation, and set the parameter of interest as the dependent variable. Each model had three covariates, namely the timing of the measurement relative to the baseline scan, the stage of ARVC at baseline (definite or non-definite), and an interaction term. As such, the models permitted the definite and non-definite AVRC groups to have separate gradients, with the *p*-value for the interaction term representing a comparison between the two gradients. Markers of MACE and disease progression were then assessed using a binary logistic regression approach. Initially, separate univariable analyses were produced for each factor of interest. Factors found to be significant on univariable analysis were then entered into a multivariable model, to identify significant independent predictors of outcomes. The Hosmer–Lemeshow test was used to test for goodness of fit for logistic regression models.

Cox regression was performed to assess markers of first MACE during follow-up. Two-sided tests were used throughout, with statistical significance defined as *p* < 0.05. All statistical analysis was performed using IBM SPSS 27.

## 3. Results

### 3.1. Clinical Characteristics

Among the 101 patients included in the study, 66 (68%) were shown to carry a known pathogenic variant associated with ARVC, as shown in (Table 1). Palpitations (n = 41 (41%)) and syncope (n = 25 (25%)) were the most common symptoms at baseline. At baseline, 22 (22%) definite patients presented with MACE, of which 20 (91%) met either minor or major imaging criteria at inclusion.

### 3.2. Serial Results

The follow-up period was similar for definite and non-definite ARVC patients, with a median of 4 years (IQR: 2–6). When patients were subdivided into stable and progressive disease groups, the majority of patients in the progressive group were male (n = 21 (91%) and syncope was the most common symptom reported (n = 14 (61%)). Additional characteristics are presented in the supplementary table (Table S1).

### 3.3. Changes over Time in Definite and Non-Definite Patients

As expected, patients with a definite diagnosis of ARVC had a more complete disease phenotype at baseline, fulfilling both minor and major imaging criteria compared to those with a non-definite diagnosis. Specifically, they showed reduced right ventricular function, RV FAC (37 (IQR: 29–46%) vs 48 (IQR: 43–54%), *p* < 0.001) and larger RV area (25 (IQR: 19–31 cm^2^) vs 17 (IQR: 15–20 cm^2^), *p* < 0.001). LV function was normal in both groups, but lower within the normal range in the definite group (LVEF (59 (IQR: 54–64%) vs 63 (IQR: 54–64%), *p* = 0.004). The presence of LGE was more common in the definite group compared to the non-definite group (n = 21 (62%) vs n = 3 (11%), *p* < 0.001) (Table 2 and Table 3).

**Figure 1:**
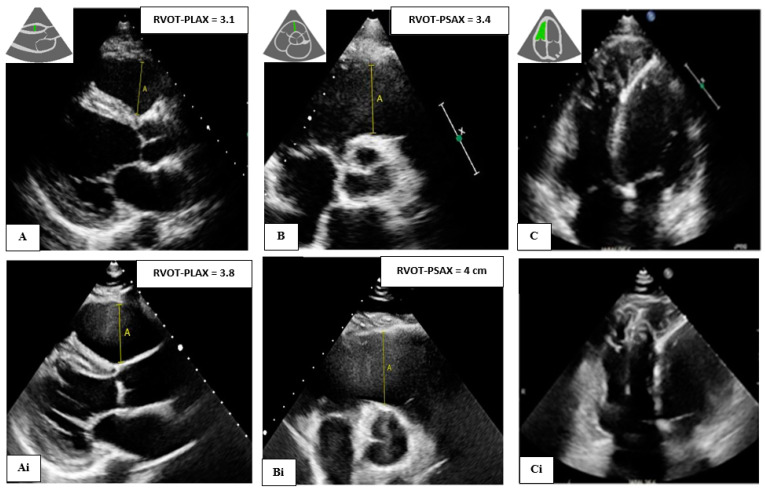
A 39-year-old male with *PKP2* pathogenic variant and definite ARVC (Top) Baseline images showing RVOT (still normal) in (**A**) parasternal longs axis (PLAX), (**B**) parasternal short axis (PSAX), and (**C**) apical four chamber view showing dilated and trabeculated right ventricle (RV). Further dilation was observed after 6 years of follow-up (**Ai**,**Bi**,**Ci**) (bottom).

**Figure 2:**
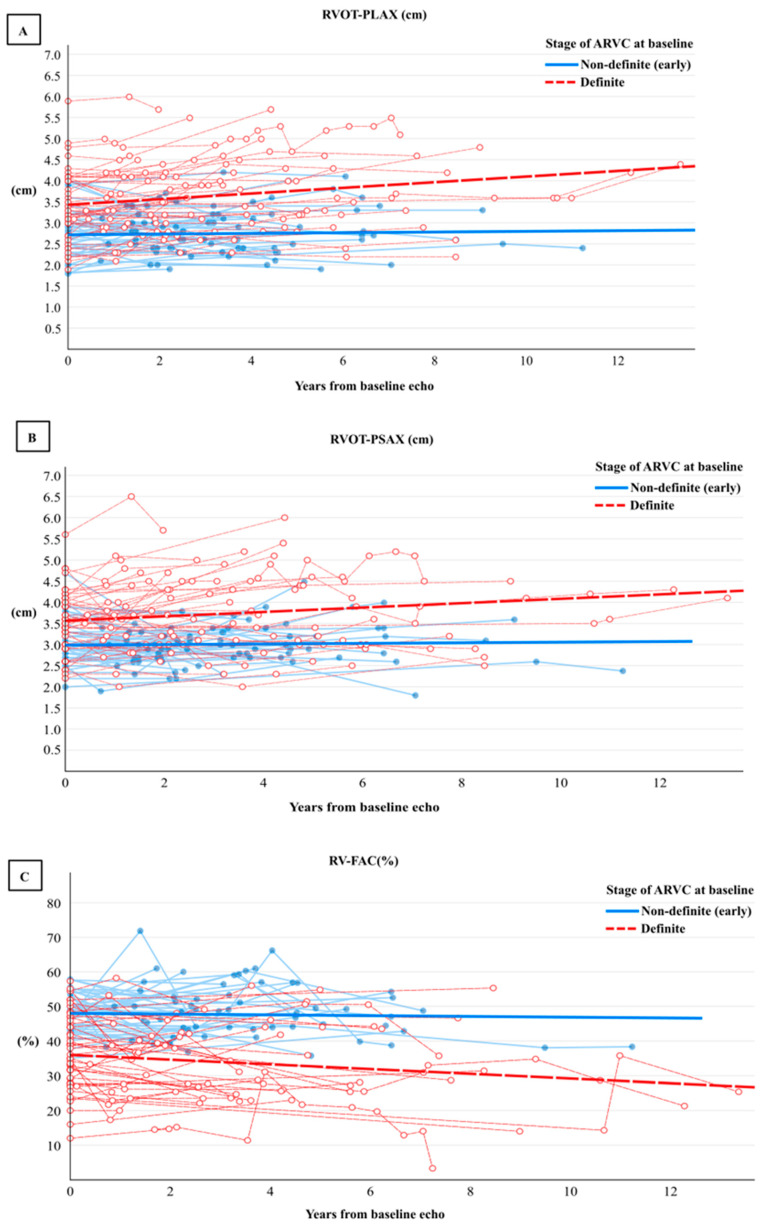
Rate of progression of right ventricular outflow tract (RVOT) diameter in PLAX (**A**), PSAX (**B**), and (**C**) right ventricle fractional area change (RV-FAC) using a generalised estimating equation approach of 306 repeated echocardiographic assessments among 101 patients with definite (red) and non-definite (early) ARVC (blue).

During the 4-year follow-up period (IQR: 2–6), the prevalence of imaging criteria increased in the definite cohort, whereas only one patient in the non-definite group developed minor imaging criteria (Table 3). Significant changes seen in the definite group included a yearly reduction in RV FAC by −1% (95% CI −1, −0.2), a yearly increase in RVOT-PLAX diameter by 0.1 cm (95% CI 0.04, 0.1) and PSAX by 0.1 cm (95% CI 0.02, 0.1), and RVEDA by 0.4 cm^2^ (95% CI 0.1, 1), (Table 4, Figure 1 and Figure 2), while LV function remained stable (Table 4).

In contrast, RV and LV parameters overall did not change significantly in the non-definite group over the 4-year period. In the non-definite group, three patients were borderline and became definite during follow-up. Two of these patients progressed electrically: the first was a 35-year-old female with a *DSP* variant who presented with an increased number of premature ventricular contractions on 24 h Holter (>500 PVC) and subepicardial late gadolinium enhancement of the LV and then gained minor repolarisation criteria (TWI in leads V4-V6). The 12-lead ECG is shown in a supplementary figure (Figure S1). The second patient was a 45-year-old male with no pathogenic variant identified who presented with minor depolarisation criteria (late potentials on SAECG) and then experienced VT during follow-up. The third patient progressed both structurally and electrically. This was a 29 year-old female with a *PKP2* variant who presented with shortness of breath, palpitations and minor ECG repolarisation (TWI in leads V1-V2) and then developed major repolarisation (TWI in V1-V5) and minor echo criteria (regional wall motion abnormality and RVOT-PLAX = 2.9 cm).

### 3.4. Changes over Time in Progressive Compared to Stable Patients

During the follow-up period, 23 (45%) definite patients progressed, while 28 (55%) patients remained stable and did not develop additional criteria. Of those patients who progressed, only two (9%) continued to participate in competitive exercise against medical advice. Those who progressed had a more severe phenotype at baseline. Comparing the baseline imaging characteristics of progressive and stable structural patients (Table S2), the former had a more dilated RV on basal (4.5 ± 0.9 vs. 3.9 ± 0.9, *p* = 0.015) and mid-dimensions (3.7 (IQR: 3.1–4.7) vs. 3.0 (IQR: 2.7–4.0), *p* = 0.037). LV function was lower by EF (56 (IQR:42–60%) vs. 61 (IQR: 59–65%), *p* = 0.003), but was still within normal limits. In the group who progressed, the annual gradient rate of the deterioration in right ventricular function measured by RV FAC was −1% (95% CI −2, −0.2); RV diameter increased in PLAX by 0.1 cm (95% CI 0.1, 0.2) and in PSAX by 0.1 cm (95% CI 0.1, 0.2), respectively. RVEDA increased by 1 cm^2^ (95% CI 0.4, 2), and LVEDV increased by 4 mL (95% CI 1.4, 6.4) (Table S3). Values for the non-progressive group over the 4-year follow-up period (IQR: 2–5) are shown in a supplementary table (Table S3).

We observed no major differences in structural progression between gene-positive and gene-negative patients with only a tendency toward more progression in RVOT-PLAX for gene-positive patients than gene-negative patients (Table S5); the baseline characteristics of both groups are presented in Table S6.

Additionally, patients with *PKP2* variant tend to show dilated RVOT in PLAX and PSAX compared to the *DSP* variant over time (Table S7). Baseline imaging characteristics between *PKP2* and *DSP* variant carriers showed no significant differences, except for a lower LV EF observed in *DSP* carriers and the presence of both RV and LV LGE (Table S8).

### 3.5. ECG Changes

At baseline, the definite ARVC patients had a more severe electrical phenotype compared to the non-definite group (Table 2). Epsilon waves and T-wave inversion from V1–V3 were only observed in the definite patients (n = 12 (24%) and n = 28 (55%), respectively; *p* < 0.001). Both the PR interval (164 ms (IQR: 142–184) and QRS duration (100 ms (IQR: 86–109)) were longer in the definite patients group compared to the non-definite group (148 ms (IQR: 134–168 ms), *p* = 0.024 and 90 ms (IQR: 84–98 ms), *p* = 0.006, respectively).

During follow-up, as shown in (Table 3), three patients in the definite group developed an Epsilon wave and six patients developed minor repolarisation criteria. In the non-definite group, two patients gained repolarisation criteria (one minor and one major) and three patients developed minor depolarisation criteria and were labelled as electrically progressed. Over time, there was a prolongation of the PR interval by 2 ms (95% CI −2.0–9) and QRS duration by 1.2 ms (95% CI −1–4) in the definite group only (Table 4).

### 3.6. Markers Associated with Disease Progression

As shown in (Table 5), male gender and LVEF were the only markers associated with disease progression in univariate analysis (OR = 12.1, *p* = 0.003 and OR = 0.9, *p* = 0.039, respectively). However, in the multivariate analysis, male gender was the only marker associated with disease progression (OR = 10, *p* = 0.010).

### 3.7. Occurrence of MACE

In total, 33 patients experienced MACE. Structural progression, TWI in V1–V3, and QT interval were strongly associated with MACE in both the univariate and multivariate analyses (Table 6). In the univariate logistic regression analysis, the odds of male gender were significantly higher in patients with MACE (OR = 5.4, *p* = 0.001), but this association did not reach statistical significance in the multivariate analysis in this cohort.

**Figure 3:**
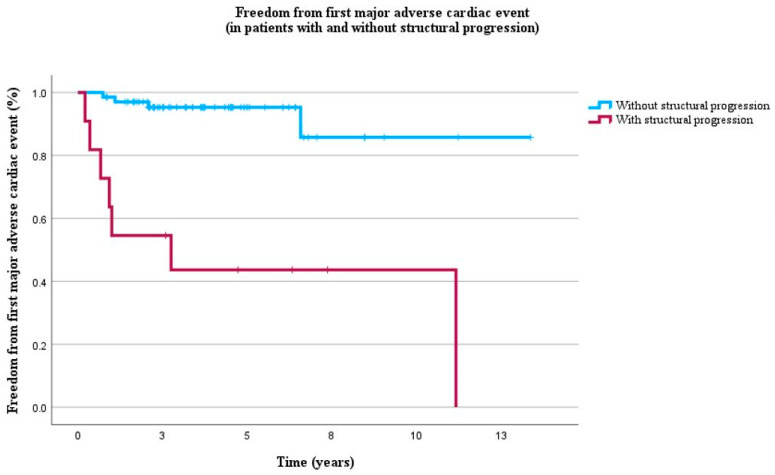
Kaplan–Meier curves of freedom from first MACE in 79 ARVC patients without history of MACE during 4 years of follow-up (IQR 2–6). Blue lines represent absence of structural progression and red lines represent presence of structural progression.

During the 4-year follow-up (IQR: 2–6), 11 patients experienced MACE for the first time, of which 7 (64%) had structural progression. Structural progression was strongly associated with first MACE (HR = 10.2, *p* = 0.005) independent of all clinical characteristics including male gender, age, pathogenic variant, electrical progression, TWI in V1–V3, and QT interval (Table 7, Figure 3).

### 3.8. Risk Estimation

Table 8 demonstrates the estimated risk of MACE between stable and progressive patients. The estimated risk of MACE within 1, 2, and 5 years was significantly higher in patients with progressive disease when compared to stable disease (*p* < 0.05). Observation in our cohort showed that patient with MACE showed a higher estimated risk in the ARVC risk score when compared to patients without MACE (*p* < 0.005), see Table 9.

## 4. Discussion

Summary of main findings. In this single-centre study of 101 patients with definite and non-definite (early) ARVC, we identified progressive changes in structure and function in more than one-third of definite ARVC patients over an average of 4 years of follow-up. These patients already had a more advanced phenotype at baseline compared both to those with definite ARVC who did not progress and also those with non-definite (early) ARVC. Significant changes in structure and function were not found in the non-definite (early) ARVC group as a whole over the period of assessment. Male gender was the only predictor of structural progression. Those who progressed were at greater risk of MACE.

Although those with progression were at greater risk of MACE events during follow-up, the size of the change or gradient of change in all parameters was small over the 4-year study period. This has two important implications: firstly, the size or gradient of change seen over four years are within the inter and intra-operator variability that one would expect on standard transthoracic echocardiography, suggesting that alternative methods are needed to track progression in the clinic, such as machine learning analysis of images and/or testing advanced imaging modalities in echocardiography and cardiac MRI [14]. Secondly, any study assessing response to therapy based on cardiovascular structure or function in ARVC should ideally aim for a longer follow-up than 4 years and use more sensitive imaging techniques or include sensitive electrical parameters.

Structural progression in echocardiography was identified best by measuring the RVOT dilatation in both the PLAX and PSAX and deterioration in RV FAC (Table 4, Figure 2 and Figure 3). These findings are consistent with a recent study [15] that included 85 patients with definite ARVC over a longer follow-up period of six years and reported an increase in RVOT diameter in the PLAX view from 35 mm (IQR, 31–39) to 37 mm (IQR, 33–41), an increase in RVOT diameter in the PSAX view from 35 mm (IQR, 31–39) to 37 mm (IQR, 34–41), and a decrease in RV FAC from 39% (IQR, 33–44%) to 34% (IQR, 24%–42%). Our data are also consistent a Norwegian study [16] that assessed structural progression in probands and family members of 144 ARVC patients over a 7-year follow-up period. The study showed that RVOT PLAX diameters increased by 0.5 mm (95% CI 0.47–0.59) and RV FAC decreased by an absolute 0.7% (95% CI 0.66–0.80). The smaller rate of change in our study in definite ARVC could be due to the shorter follow-up period and the lower number of definite patients. The absence of any significant progression in LV structure or function in our cohort is in line with findings from a multinational retrospective study [17] in which 315 echocardiographic examinations were analysed over a 10-year follow-up period. Overall, our findings suggest that a follow-up should ideally be longer than 4 years if including early ARVC patients when designing intervention or therapeutic studies in ARVC based on structural changes in echocardiography.

Male gender was confirmed as a marker associated with structural progression in this analysis. This is consistent with previous studies [18]. This could be explained by effects of strenuous exercise, sexual dimorphism in rate of desmosomal degradation, or metabolic effects of elevated serum testosterone levels in male patients [19,20].

In our study, no overall changes in conventional echocardiography parameters were observed in the non-definite (early) patients over the period of follow-up. This finding is consistent with a previous smaller study [21] that followed up 34 early patients (defined as possible or borderline ARVC) over seven years and also found no significant disease progression using conventional echocardiography parameters. In their study, deformation imaging unmasked RV basal mechanical deterioration in 14 (39%) early patients, which suggests that echocardiographic deformation imaging may have a superior role over conventional parameters during the early stages of the disease. The lack of change in non-definite (early) patients is in contrast to a Norwegian study [16] which identified structural progression not only in definite probands but also in gene-positive, phenotype-negative family members. This difference could be due to several reasons. It is possible that disease progression may be slow in early stages or vary in rate of change. Also, in the ICC clinic at UHB, Birmingham, UK, it was standard clinical practice during the period of the study to suggest to patients with non-definite (early) disease to reduce their physical activity to avoid progression and this may have confounded our outcome. Several reports [22,23,24] have confirmed that intense exercise accelerates the disease progression and increases the risk of ventricular arrhythmias in ARVC patients.

Recently, the influence of exercise restriction on ventricular function in 27 ARVC athletes has been investigated [25]. During follow-up, n = 15 (56%) stopped intensive exercise and n = 12 (44%) continued exercising. It was shown that the group which continued exercising demonstrated a decreasing trend in LV ejection fraction assessed by echocardiography. On the contrary, LVEF remained stable in the group which stopped intensive exercise during follow-up. Even stricter advice was given to patients with definite ARVC who often showed a history of exercise and, overall, still progressed in our cohort. This may suggest exercise restriction to be useful already in early disease stages, but more focused and prospective studies on this aspect are needed. Larger studies following up non-definite (early) patients will need to study cohorts over longer follow-up duration, particularly since conventional echocardiographic criteria lack sensitivity in ARVC diagnosis as suggested by a recent validation study [26].

In our cohort, T-wave inversion in V1–V3 was observed in (28%). T-wave inversion is reported in competitive athletes, but it has been observed that T-wave inversion extending beyond lead V3 is more common in ARVC patients than in athletes and the general population [27]. Also, it has been shown that T-wave inversion in V1–V2 does not convey the risk of SCD and it could be considered as a normal observation, especially in younger people [28].

Our study showed that structural progression was strongly associated with occurrence of MACE, independent of clinical and electrical markers (Table 7, Figure 3). This finding is in line with a previous study that reported associations between structural progression and VA in patients with definite ARVC diagnosis [16]. These results suggest that the detection of structural progression, as determined by both ventricular function and structure, is critical in identifying patients who are at high risk of experiencing MACE.

## 5. Clinical Implications

Our findings demonstrate that individuals with structural progression had a higher risk of MACE, despite that structural and functional alterations were perceived as minor. Definite ARVC cases showed notable disease progression when compared to non-definite (early). This raises the need for personalised surveillance and treatment approaches, especially in definite patients. Additionally, studies should take into account test–retest reproducibility and the duration of the follow-up. The potential including structural progression in the ARVC risk calculator after baseline could be examined.

## 6. Limitations

This was a repeated-measure, retrospective, longitudinal study with its associated limitations, one of which is the risk of selection bias. Data in this study were only from the patients that had follow ups. Data were unavailable for 24 out of the 168 patients. Patients were recruited from a single centre with various follow-up intervals. The analysis was limited by the small number of events and patients with a rare disease. SAECG and 24-Holter monitoring were not available for all patients. The outcomes could be different when assessing other populations with different genetic and environmental factors. Data from this registry should ideally be included into multicentre and international registries in the future. This study focused on serial echocardiography as only a fraction of patients received CMR and other investigations (n = 29).

## 7. Conclusions

Our study found that structural and functional progression occurred in over one-third of definite ARVC patients during four years of follow-up. Although the size of the changes identified was small, those patients who demonstrated structural progression were at higher risk of MACE, indicating that monitoring the rate of progression may guide future risk stratification and therapy. Male gender predicted structural progression during follow-up. No changes were noted overall in those with non-definite (early) ARVC in our centre during a mean follow-up of four years.

## Figures and Tables

**Table 1:** Clinical and demographic biomarkers of the study cohort.

Demographic	Total (n = 101)	Non-Definite (n = 50)	Definite (n = 51)	*p* Value
Age (years)	39 (27, 53)	36 (23, 53)	41 (30, 56)	0.124
Male sex, n (%)	58 (57%)	24 (48%)	34 (67%)	0.071
BSA (m^2^)	1.9 (1.7, 2.0)	1.9 (1.7, 2.0)	1.9 (1.7, 2.1)	0.401
^a^ Identification of a pathogenic variant, n (%)	66/97 (68%)	32/50 (64%)	34/47 (72%)	0.394
**Symptoms**				
Palpitation, n (%)	41 (41%)	11 (22%)	30 (59%)	**<0.001**
Syncope, n (%)	25 (25%)	4 (8%)	21 (41%)	**<0.001**
**History of sport**				
Competitive sport, n (%)	21 (23%)	6 (14%)	15 (31%)	0.072
Non-competitive sport, n (%)	22 (24%)	11 (26%)	11 (23%)	
**Medication**				
Statins, n (%)	3 (3%)	0 (0%)	3 (6%)	0.243
Anticoagulant, n (%)	6 (6%)	0 (0%)	6 (12%)	**0.027**
Antiarrhythmic drugs, n (%)	16 (16%)	1 (2%)	15 (29%)	**<0.001**
Beta blocker, n (%)	23 (23%)	1 (2%)	22 (43%)	**<0.001**
**Biomarkers**				
NT-proBNP (ng/L)	315 (132, 498)	58 (42, 169)	438 (231, 618)	**0.006**

Abbreviations: BSA: body surface area; NT-proBNP: B-type natriuretic peptide. Data are reported as median (IQR), with *p*-values from Mann-Whitney U tests; or as n: number (%), with *p*-values from Fisher’s exact tests. **^a^** Genetic testing was performed in 97 patients.

**Table 2:** Electrical and imaging characteristics of definite and non-definite ARVC.

Baseline ECG	Total (n = 101)	Non-Definite (n = 50)	Definite (n = 51)	*p* Value
Heart rate (bpm)	64 (56, 73)	66 (57, 73)	59 (53, 78)	0.308
PR interval (ms)	154 (138, 176)	148 (134, 168)	160 (142, 184)	**0.024**
QRS duration (ms)	94 (86, 104)	90 (84, 98)	100 (86, 109)	**0.006**
QT (ms)	409 ± 39	398 ± 31	419 ± 43	**0.004**
QTc (ms)	418 ± 26	412 ± 22	424 ± 29	**0.025**
**Depolarisation criteria**				
Major criteria, n (%), Epsilon wave in the right precordial leads (V1–V3), n (%)	12 (12%)	0 (0%)	12 (24%)	**<0.001**
Minor criteria, n (%) Signal-averaged ECG with late potential (if QRS on standard surface < 110 ms), n (%)	44 (51%)	19 (40%)	25 (64%)	**0.033**
**Repolarisation criteria**				
Major criteria, n (%), TWI in right precordial leads (V1, V2 and V3), n (%)	28 (28%)	0 (0%)	28 (55%)	**<0.001**
^a^ Any minor criteria, n (%), TWI in leads V1 and V2 or in V4, V5, and V6, TWI in leads V1, V2, V3, and V4 with RBBB, n (%)	24 (24%)	2 (4%)	22 (43%)	**<0.001**
^b^ >500 PVC/24 h (Holter), n (%)	22 (48%)	3 (16%)	19 (70%)	**<0.001**
**Echo data**				
RVOT-PLAX (cm)	3.0 (2.5, 3.5)	2.7 (2.4, 3.0)	3.3 (2.7, 4.0)	**<0.001**
RVOT-PSAX (cm)	3.2 (2.9, 3.7)	3.0 (2.7, 3.3)	3.5 (3.0, 4.0)	**<0.001**
RV-base (cm)	3.5 (3.1, 4.4)	3.2 (3.0, 3.6)	4.1 (3.3, 4.8)	**<0.001**
RV-mid (cm)	3.0 (2.6, 3.5)	2.8 (2.5, 3.2)	3.4 (2.7, 4.2)	**<0.001**
RV-EDA (cm^2^)	19 (16, 26)	17 (15, 20)	25 (19, 31)	**<0.001**
RV-FAC (%)	44 (35, 50)	48 (43, 54)	37 (29, 46)	**<0.001**
LV-EDV (mL)	92 ± 30	92 ± 28	93 ± 32	0.948
LV-EF (%)	61 (57, 66)	63 (58, 67)	59 (54, 64)	**0.004**
**^c^ CMR data**				
RV-EDV (mL)	173 (144, 219)	163 (137, 187)	191 (150, 232)	**0.048**
RV-EF (%)	51 (39, 57)	56 (51, 60)	43 (28, 50)	**<0.001**
LV-EDV (mL)	155 (122, 190)	157 (129, 188)	154 (121, 203)	0.632
LV-EF (%)	61 (54, 69)	64 (59, 69)	59 (46, 69)	0.211
^d^ LGE present, n (%)	24 (39%)	3 (11%)	21 (62%)	**<0.001**
RV and LV LGE, n (%)	11 (18%)	0 (0%)	11 (32%)	**<0.001**
LV—specific LGE, n (%)	4 (7%)	2 (7%)	2 (6%)	1.000
RV—specific LGE, n (%)	9 (15%)	1 (4%)	8 (24%)	**0.036**

Abbreviations: TWI: T-wave inversion; RBB: right bundle branch block; PVC: premature ventricular contractions; RVOT PLAX: right ventricular outflow tract parasternal long axis; RVOT PSAX: right ventricular outflow tract parasternal short axis; RVEDA: right ventricular end diastolic area; RVFAC: right ventricular fractional area change; LVEDD: left ventricular end-diastolic volume; LVEF: left ventricular ejection fraction; LGE: late gadolinium enhancement. Data are reported as mean ± SD, with *p*-values from independent samples t-tests; median (IQR), with *p*-values from Mann-Whitney U tests; or as n (%), with *p* values from Fisher’s exact tests. ^a^ SAECG was performed in 86 patients. ^b^ 24 Holter monitoring was performed in 46 patients. ^c^ CMR was performed in 64 patients (28 non-definite and 36 definite patients). ^d^ LGE was assessed in 61 patients.

**Table 3:** 2010 Task Force Criteria and major adverse cardiac events at baseline and during follow up.

	Definite (n = 51)				Non Definite (n = 50)			
	At Baseline	During Follow-Up	*p* Value	N	At Baseline	During Follow-Up	*p* Value	N
Major echo criteria, n (%)	24 (47%)	33 (65%)	0.004	51	0 (0%)	0 (0%)	NA	50
Minor echo criteria, n (%)	2 (4%)	3 (6%)	1.000	51	0 (0%)	1 (2%)	1.000	50
Major CMR criteria, n (%)	3 (38%)	4 (50%)	1.000	8	0 (%)	0 (0%)	NA	6
Minor CMR criteria, n (%)	1 (13%)	8 (100%)	1.000	8	0 (%)	0 (0%)	NA	6
**Depolarisation criteria**								
Major criteria, n (%), Epsilon wave in the right precordial leads (V1–V3), n (%)	12 (24%)	15 (29%)	0.250	51	0 (%)	0 (0%)	NA	50
Minor criteria, n (%) Signal-averaged ECG with late potential (if QRS on standard surface <110 ms), n (%)	15 (58%)	17 (65%)	0.500	26	15 (41%)	17 (46%)	0.687	37
**Repolarisation criteria**								
Major criteria, n (%), TWI in right precordial leads (V1, V2 and V3), n (%)	28 (55%)	29 (57%)	1.000	51	0 (0%)	1 (2%)	1.000	50
Any minor criteria, n (%), TWI in leads V1 and V2 or in V4, V5, and V6, TWI in leads V1, V2, V3, and V4 with RBBB, n (%)	22 (43%)	28 (55%)	0.070	51	2 (4%)	3 (6%)	1.000	50
Ventricular fibrillation (VF), n (%)	8 (16%)	0 (0%)	**0.008**	51	0 (%)	0 (0%)	NA	50
Sustained ventricular tachycardia (VT), n (%)	12 (24%)	4 (8%)	0.077	51	0 (%)	1 (2%)	1.000	50
Heart failure (HF), n (%)	2 (4%)	5 (10%)	**0.039**	51	0 (%)	0 (0%)	NA	50
ICD implanted, n (%)	12 (24%)	21 (41%)	0.164	51	0 (%)	3 (6%)	0.250	50
ICD therapy (shock/ATP), n (%)	5 (10%)	12 (24%)	0.143	51	0 (0)	1 (2)	1.000	50

Abbreviations: CMR: cardiac magnetic resonance; TWI: T-wave inversion; RBB: right bundle branch block; PVC: premature ventricular contractions. Data are reported or as n: number (%), with *p* values from McNemar’s test.

**Table 4:** Echocardiography and ECG changes over time in definite and non-definite ARVC patients.

Markers	Gradient per Year Measured over 4 Years				Interaction *p* Value
	Non-Definite at Baseline (n = 50)		Definite at Baseline (n = 51)		
	Statistics (95%CI)	*p*-Value	Statistics (95%CI)	*p*-Value	
RVOT-PLAX (cm)	0.01 (−0.02, 0.03)	0.540	0.1 (0.04, 0.1)	**<0.001**	**0.002**
RVOT-PSAX (cm)	0.01 (−0.03, 0.04)	0.688	0.1 (0.02, 0.1)	**0.001**	**0.042**
RV-base (cm)	0.02 (−0.01, 0.04)	0.248	0.04 (0.00, 0.1)	**0.032**	0.313
RV-mid (cm)	0.00 (−0.03, 0.03)	0.901	0.01 (−0.03, 0.1)	0.565	0.714
RV-EDA (cm^2^)	0.1 (−0.3, 0.4)	0.709	0.4 (0.1, 1)	**0.024**	0.168
RV-FAC (%)	−0.11 (−1, 1)	0.719	−1 (−1, −0.2)	**0.008**	0.161
LV-EDV (mL)	1 (−1, 3)	0.335	1.2 (−1, 3)	0.212	0.859
LV-EF (%)	−0.1 (−0.4, 0.3)	0.648	0.14 (−0.4, 1)	0.589	0.481
HR (bpm)	−0.1 (−3, 2.0)	0.943	0.2 (−4, 3)	0.739	0.739
PR interval (ms)	0.4 (−4, 3)	0.858	2.0 (−2, 9)	**0.026**	**0.026**
QRS dur (ms)	1 (−1, 2)	0.184	1.2 (−1, 4)	**0.013**	**0.013**

Abbreviations: RVOT-PLAX: right ventricular outflow tract parasternal long axis; RVOT-PSAX: right ventricular outflow tract parasternal short axis; RV-EDA: right ventricular end diastolic area; RV-FAC: right ventricular fractional area change; LV-EDV: left ventricular end-diastolic volume; LV-EF: left ventricular ejection fraction; HR: heart rate. Longitudinal trends were analysed using a generalised estimating equation approach.

**Table 5:** Marker at baseline associated with disease progression.

	Disease Progression		Univariable Analysis		Multivariable Analysis	
Marker	Stable (n = 28)	Progressed (n = 23)	OR (95%CI)	*p* Value	OR (95%CI)	*p* Value
Sex, n (%)						
Female	15/17 (88%)	2/17 (12%)	-	-		
Male	13/34 (38%)	21/34 (62%)	12.1 (2.4–61.8)	**0.003**	10 (1.7–57.3)	**0.010**
LGE, n (%)						
Absent	9/13 (69%)	4/13 (31%)	-	-	-	-
Present	11/21 (52%)	10/21 (48%)	2.0 (0.5–8.8)	0.335		
Competitive sport, n (%),						
No	21/33 (64%)	12/33 (36%)	-			
Yes	5/15 (33%)	10/15 (67%)	-			
			3.5 (1.0–12.7)	0.056		
Major criteria, n (%), Epsilon wave in the right precordial leads (V1–V3), n (%)						
Absent	24/39 (62%)	15/39 (38%)	-		-	-
Present	4/12(33%)	8/12 (67%)	-			
			3.2 (0.8–12.5)	0.094		
Major criteria, n (%), TWI in right precordial leads (V1, V2 and V3), n (%)						
Absent	10/23 (43%)	13/23 (57%)	-		-	-
Present	18/28 (64%)	10/28 (36%)	-			
			0.4 (0.1–1.3)	0.140		
RVOT-PLAX (cm), median (IQR)	3.2 (2.6–4.0)	3.3 (3.0–4.1)	1.2 (0.6–2.4)	0.578	-	-
RVOT-PSAX (cm), median (IQR)	3.4 (3.0–4.0)	3.5 (3.0–3.9)	0.9 (0.4–2.0)	0.804	-	-
RV-FAC (%), median (IQR)	44 (29–48)	35 (29–40)	1.0 (0.9–1.0)	0.336	-	-
LV-EF (%), median (IQR)	61 (59–65)	56 (42–60)	0.9 (0.9–1.0)	**0.039**	0.9 (0.9–1.0)	0.079

Abbreviations: LGE: late gadolinium enhancement; RVOT-PLAX: right ventricular outflow tract parasternal long axis; RVOT-PSAX: right ventricular outflow tract parasternal short axis; RV-FAC: right ventricular fractional area change; LV-EF: left ventricular ejection fraction. Markers of disease progression were assessed using a binary logistic regression approach.

**Table 6:** Markers associated with MACE based on univariable and multivariable logistic regression analyses in patients with MACE at entry and during follow-up.

	Overall MACE		Univariable Analysis		Multivariable Analysis	
Marker	MACE (n = 33)	No MACE (n = 68)	OR (95% CI)	*p* Value	OR (95% CI)	*p* Value
Sex, n (%)						
Female	6/43 (14%)	37/43 (86%)	-		-	-
Male	27/58 (47%)	31/58 (53%)	-			
			5.4 (2.0–14.7)	**0.001**	3.4 (1.0–12.1)	0.055
Structural progression, n (%)						
Absent	13/77 (17%)	64/77 (83%)	-			
Present	20/24 (83%)	4/24 (17%)	-			
			24.6 (7.2–84)	**<0.001**	21.7 (5.1–92.1)	<0.001
ECG electrical progression, n (%)						
Absent	23/73 (32%)	50/73 (68%)	-		-	-
Present	10/28 (36%)	18/28 (64%)	-			
			1.2 (0.5–3.0)	0.687		
Pathogenic variant, n (%)						
Absent	12/31 (39%)	19/31 (61%)	-		-	-
Present	19/66 (29%)	47/66 (71%)	-			
			0.6 (0.3–1.6)	0.330		
Age (years),	41 (31–55)	37 (25–53)	1.0 (1.0–1.0)	0.269	-	-
median (IQR)						
TWI in V1-V3, n (%)						
Absent	16/73 (22%)	57/73 (78%)	5.5 (2.2–14.1)	<0.001	7.1 (1.9–26.2)	**0.003**
Present	17/28 (61%)	11/28 (39%)				
QT interval,	430 (398–462)	401 (376–422)	1.0 (1.0–1.0)	0.002	1.0 (1.0–1.0)	**0.034**
median (IQR)						

Abbreviations: CI: confidence interval; OR: odds ratio. Markers of MACE were assessed using a binary logistic regression approach.

**Table 7:** Markers of first MACE occurring during follow up in 79 ARVC patients (excluding patients with a history of MACE).

Markers	First MACE during Follow-Up			
	MACE (n = 11)	No MACE (n = 68)	HR (95% CI)	*p* Value
**Age (years), median (IQR)**	41 (29–50)	37 (25–53)	1.0 (1.0–1.0)	0.993
**Male sex, n (%)**	8 (73%)	31 (64%)	0.9 (0.2–5.3)	0.939
**Pathogenic variant, n (%)**	6 (55%)	47 (71%)	0.4 (0.1–1.7)	0.199
**Structural progression, n (%)**	7 (64%)	4 (6%)	10.2 (2.0–52.0)	**0.005**
**ECG electrical progression, n (%)**	4 (36%)	18 (26%)	1.7 (0.4–7.6)	0.466
**QT interval, median (IQR)**	420 (400–460)	401 (376–422)	1.0 (1.0–1.0)	0.660
**TWI in V1-V3, n (%)**	3 (27%)	11 (16%)	1.7 (0.3–9.1)	0.511

Abbreviations: CI: confidence interval; HR: hazard ratio. First MACE during follow-up was assessed using multivariable Cox regression.

**Table 8:** Estimated risk of MACE from the ARVC Risk Calculator in patients with progressive and stable disease.

Estimated Risk of MACE from ARVC Risk Calculator	Stable Disease (n = 22/28)	Progressive Structural Disease, (n = 7/23)	*p*-Value
Estimated risk within 1 year (%)	3 (2–6)	16 (8–28)	**0.001**
Estimated risk within 2 years (%)	5 (3–10)	24 (13–42)	**0.001**
Estimated risk within 5 years (%)	9 (5–16)	37 (21–59)	**0.002**

For each patient, the risk of MACE within 1, 2, and 5 years of the baseline assessment was estimated using the ARVC Risk Calculator. Patients were then divided into two groups based on whether they had stable or progressive structural disease during the follow-up period, for which the median (IQR) of the estimated risk are reported and compared using the Mann–Whitney U test; bold *p*-values are significant at *p* < 0.05.

**Table 9:** Estimated risk of MACE from the ARVC Risk Calculator in patients with a definite ARVC diagnosis with and without MACE.

Estimated Risk of MACE from ARVC Risk Calculator	MACE, (n = 5/51)	No MACE, (n = 24/51)	*p*-Value
Estimated risk within 1 year (%)	19 (16–20)	3 (2–7)	**0.002**
Estimated risk within 2 years (%)	28 (25–31)	6 (3–11)	**0.002**
Estimated risk within 5 years (%)	43 (38–46)	9 (5–18)	**0.003**

For each patient, the risk of MACE within 1, 2, and 5 years of the baseline assessment was estimated using the ARVC Risk Calculator. Patients were then divided into two groups based on whether they had MACE during the follow-up period, for which the median (IQR) of the estimated risk are reported and compared using the Mann–Whitney U test; bold *p*-values are significant at *p* < 0.05.

## Data Availability

The anonymised datasets used and/or analysed during the current study are available from the corresponding author upon reasonable request.

## Supplementary Materials

The following supporting information can be downloaded at: https://www.mdpi.com/article/10.3390/biomedicines12020328/s1, Table S1: Clinical and demographic biomarkers of stable and progressive structural disease; Table S2: Electrical and imaging characteristics of stable and progressive structural disease; Table S3: Structural and electrical progression over time in stable and progressive structural disease; Table S4: 2010 TFC and Major adverse cardiac events at baseline and during follow-up in stable and progressive structural disease; Table S5: Structural progression over time between gene positive and gene negative patients; Table S6: Clinical and demographic biomarkers of patients with and without pathogenic variant; Table S7. Structural progression over time between patients with *PKP2* and *DSP* variants; Table S8. Baseline imaging characteristics between patients with *PKP2* and *DSP* variants; Table S9. Definite ARVC patients (n = 15) with Epsilon wave classified as minor criterion per 2020 Criteria”. Figure S1. Original baseline and follow up ECG of a pathogenic variant carrier.

## Abbreviations

ARVC: arrhythmogenic right ventricular cardiomyopathy; VA: ventricular arrhythmias; SCD: sudden cardiac death; VF: ventricular fibrillation; MACE: major adverse cardiac even; HF: heart failure; CMR: cardiovascular magnetic resonance; ECG: electrocardiogram; SAECG: signal-averaged ECG; TWI: T-wave inversion; RBB: right bundle branch block; PVC: premature ventricular contractions; ICD: implantable cardioverter-defibrillator; LGE: late gadolinium enhancement; LV: left ventricle; RV: right ventricle; SCD: sudden cardiac death; TFC: task force criteria; RVOT: right ventricle outflow tract; PLAX; parasternal long axis; PSAX: parasternal short axis; FAC: fractional area change; RVEDA: right ventricular end diastolic area; RVFAC: right ventricular fractional area change; LVEDD: left ventricular end-diastolic volume; LVEF: left ventricular ejection fraction.
