# Supplementary material for: Structural Progression in Patients with Definite and Non-Definite Arrhythmogenic Right Ventricular Cardiomyopathy and Risk of Major Adverse Cardiac Events
Source: Biomedicines. 2024 Jan 31;12(2):328. doi: 10.3390/biomedicines12020328 (PMC10886648; doi:10.3390/biomedicines12020328)
Supplement: Supplementary file 1 [file biomedicines-12-00328-s001.zip › biomedicines-2709748-supplementary.pdf]

Supplementary Online Content

Table S1. Clinical and demographic biomarkers of stable and progressive structural disease

Table S2. Electrical and imaging characteristics of stable and progressive structural disease

Table S3. Structural and electrical progression over time in stable and progressive structural disease

Table S4. 2010 TFC and Major adverse cardiac events at baseline and during follow-up in stable and progressive structural disease

Table S5. Structural progression over time between gene positive and gene negative patients

Table S6. Clinical and demographic biomarkers of patients with and without pathogenic variant

Table S7. Structural progression over time between patients with *PKP2* and *DSP* variants

Table S8. Baseline imaging characteristics between patients with *PKP2* and *DSP* variants

Table S9. Definite ARVC patients (n=15) with Epsilon wave classified as minor criterion per 2020 Criteria"

This supplementary material has been provided by the authors to give readers additional information about their work.

Table S1. Clinical and demographic biomarkers of stable and progressive structural disease

**Clinical characteristics at baseline**	**Stable disease (n = 28)**	**Progressive structural disease (n = 23)**	**p value**
Age (years), n (%)	42 ± 14	43 ± 18	0.834
Male gender, n (%)	13 (46%)	21 (91%)	**0.001**
BSA (m^2)	1.9 ± 0.3	2.0 ± 0.2	0.143
**Ethnicity**			
White, n (%)	17 (61%)	15 (65%)	0.291
Asian, n (%)	3 (11%)	5 (22%)	
Black, n (%)	2 (7%)	2 (9%)	
Non-specified/mixed, n (%)	6 (21%)	1 (4%)	
Identification of a pathogenic variant, n (%)	20 (80%)	14 (64%)	0.328
**Symptoms**			
Palpitation, n (%)	14 (50%)	16 (70%)	0.253
Syncope, n (%)	7 (25%)	14 (61%)	**0.012**
**History of sport**			
Competitive sport, n (%)	5 (19%)	10 (45%)	0.113
Non-competitive sport, n (%)	8 (31%)	3 (14%)	
**Biomarkers**			
NT-proBNP (ng/L)	491 (132, 753)	431 (273, 478)	0.943

Abbreviations: BSA: body surface area; NT-proBNP: N-terminal pro-B-type natriuretic peptide. Data are reported as mean ±SD, with p-values from independent samples t-tests; median (IQR), with p-values from Mann-Whitney U tests; or as n: number (%), with p-values from Fisher's exact tests.

Table S2. Electrical and imaging characteristics of stable and progressive structural disease

Baseline ECG	Stable disease (n = 28)	Progressive structural disease (n = 23)	p value
Heart rate (bpm)	65 (54, 76)	59 (53, 79)	0.917
PR interval (ms)	159 (138, 183)	166 (148, 190)	0.353
QRS duration (ms)	98 (86, 106)	104 (88, 114)	0.244
QT (ms)	418 ± 43	422 ± 43	0.745
QTc (ms)	426 ± 31	422 ± 27	0.697
P axes	56 ± 16	50 ± 18	0.221
QRS axes	39 ± 44	34 ± 59	0.732
T axes	27 (-10, 52)	15 (-7, 47)	0.532
Major ECG depolarisation, n (%)	4 (14%)	8 (35%)	0.107
Minor ECG depolarisation, n (%)	14 (64%)	11 (65%)	1.000
Major ECG repolarisation, n (%)	18 (64%)	10 (36%)	0.166
Minor ECG repolarisation, n (%)	10 (36%)	12 (52%)	0.269
>500 PVC / 24 hours (Holter), n (%)	9 (64%)	10 (77%)	0.678
**Echo**			
RVOT-PLAX (cm)	3.3 ± 0.9	3.4 ± 0.8	0.586
RVOT-PSAX (cm)	3.5 ± 0.8	3.5 ± 0.7	0.809
RV-base (cm)	3.9 ± 0.9	4.5 ± 0.9	**0.015**
RV-mid (cm)	3.0 (2.7, 4.0)	3.7 (3.1, 4.7)	**0.037**
RV-EDA (cm^2)	22 (17, 28)	28 (19, 33)	0.081
RV-FAC (%)	39 ± 13	35 ± 8	0.343
LV-EDV (ml)	91 ± 28	94 ± 36	0.793
LV-EF (%)	61 (59, 65)	56 (42, 60)	**0.003**
**CMR**			
RV-EDV (ml)	188 (128, 228)	194 (159, 255)	0.229
RV-EF (%)	44 ± 17	34 ± 10	**0.038**
LV-EDV (ml)	132 (120, 187)	156 (127, 219)	0.282
LV-EF (%)	61 ± 18	52 ± 14	0.106
LGE present, n (%)	11 (55%)	10 (71%)	0.477

Abbreviations: HR: heart rate; premature ventricular contractions; RVOT PLAX: right ventricular outflow tract parasternal long axis; RVOT PSAX: right ventricular outflow tract parasternal short axis; RVEDA: right ventricular end diastolic area; RVFAC: right ventricular fractional area change; LVEDV: left ventricular end-diastolic volume; LV EF: left ventricular ejection fraction; LGE: late gadolinium enhancement. Data are reported as mean±SD, with p-values from independent samples t-tests; median (IQR), with p-values from Mann-Whitney tests; or n: number (%), with p-values from Fisher's exact tests.

Table S3. Structural and electrical progression over time in stable and progressive structural disease

Markers	Gradient per year				Interaction p value
	**Stable disease (n = 28)**		**Progressive structural disease (n = 23)**		
	Statistics (95%CI)	p-value	Statistics (95%CI)	p-value	
RVOT-PLAX (cm)	0.04 (0.01, 0.1)	**0.003**	0.1 (1, 0.2)	**<0.001**	**0.001**
RVOT-PSAX (cm)	0.03 (-0.00, 0.1)	0.092	0.12 (0.1, 0.2)	**<0.001**	**0.004**
RV-base (cm)	0.02 (-0.02, 0.1)	0.378	0.1 (-0.01, 0.12)	0.069	0.319
RV-mid (cm)	-0.01 (-0.1, 0.1)	0.812	0.04 (-0.01, 0.1)	0.139	0.213
RV-EDA (cm^2)	0.11 (-0.3, 1)	0.620	1 (0.4, 2)	**0.001**	**0.018**
RV-FAC (%)	-0.5 (-1.10, 0.13)	0.124	-1 (-2, -0.2)	**0.011**	0.336
LV-EDV (ml)	-2 (-4, -0.1)	**0.042**	4 (1.4, 6.4)	**0.002**	**<0.001**
LV-EF (%)	-0.2 (-0.72, 0.32)	0.451	1 (-0.30, 2)	0.183	0.126
HR (bpm)	0.5 (-3.4, 4.0)	0.682	0.0 (-4, 3)	0.394	0.320
PR interval (ms)	1.1 (-4.2, 6)	0.554	5 (0.3, 12)	**0.011**	0.130
QRS dur (ms)	1 (-2, 3)	0.439	2 (0.0, 8)	**0.005**	0.087

Abbreviations: RVOT PLAX: right ventricular outflow tract parasternal long axis; RVOT PSAX: right ventricular outflow tract parasternal short axis; RVEDA: right ventricular end diastolic area; RVFAC: right ventricular fractional area change; LVEDV: left ventricular end-diastolic volume; LVEF: left ventricular ejection fraction; HR: heart rate. Longitudinal trends were analysed using a generalized estimating equation approach.

Table S4. 2010 TFC and Major adverse cardiac events at baseline and during follow-up in stable and progressive structural disease

	Stable disease (n = 28)				Progressive structural disease (n =23)			
	**At baseline**	**During follow-up**	**p value**	***n**	**At baseline**	**During follow-up**	**p value**	***n**
Major ECG depolarisation, n (%)	4 (14%)	5 (18%)	1.000	28	8 (35%)	10 (43%)	0.500	23
Minor ECG depolarisation, n (%)	10 (63%)	11 (69%)	1.000	16	5 (50%)	6 (60%)	1.000	10
Major ECG repolarisation, n (%)	18 (64%)	18 (64%)	1.000	28	10 (43%)	11 (48%)	1.000	23
Minor ECG repolarisation, n (%)	10 (36%)	14 (50%)	0.125	28	12 (52%)	14 (61%)	0.625	23
>500 PVC / 24 hours (Holter)	6 (67%)	5 (56%)	1.000	9	5 (83%)	5 (83%)	1.000	6
Ventricular fibrillation, n (%), n (%)	2 (7%)	0 (100%)	0.500	28	6 (26%)	0 (0%)	**0.031**	23
Sustained ventricular tachycardia, n (%)	7 (25%)	1 (4%)	0.070	28	5 (22%)	3 (13%)	0.727	23
Heart failure, n (%)	2 (7%)	1 (4%)	1.000	28	0 (0%)	4 (17%)	0.125	23
ICD implanted, n (%)	4 (14%)	11 (39%)	0.118	28	8 (35%)	10 (43%)	0.815	23
ICD therapy (shock/ATP), n (%)	1 (4%)	3 (11%)	0.625	28	4 (17%)	9 (39%)	0.267	23

*Data are reported as n: number (%), with p-values from McNemar's test. *n represent the number of cases present at baseline and during follow-up.*

Table S5. Structural progression over time between gene positive and gene negative patients

Markers	Gradient per year measured over 4 years				Interaction p-value
	**Patients with pathogenic variants (n = 66)**		**Patients with no pathogenic variants (n = 31)**		
	Statistics (95% CI)	p-value	Statistics (95% CI)	p-value	
RVOT-PLAX (cm)	0.04 (0.02, 0.1)	**<0.001**	0.1 (0.01, 0.1)	**0.015**	0.721
RVOT-PSAX (cm)	0.03 (0.01, 0.1)	0.004	0.04 (-0.01, 0.10)	0.122	0.722
RV-FAC (%)	-0.4 (-1, 0.1)	0.106	-1 (-2, 0.3)	0.175	0.619
LV-EF (%)	-0.04 (-0.5, 0.4)	0.834	0.2 (-1, 1)	0.656	0.624

Abbreviations: RVOT PLAX: right ventricular outflow tract parasternal long axis; RVOT PSAX: right ventricular outflow tract parasternal short axis; RVFAC: right ventricular fractional area change; LVEF: left ventricular ejection fraction. Longitudinal trends were analysed using a generalized estimating equation approach.

Table S6. Clinical and demographic biomarkers of patients with and without pathogenic variant

Demographic	Total (n= 97)	Patients with pathogenic variants (n = 66)	Patients with no pathogenic variants (n = 31)	p value
Age (years)	38 (27, 53)	39 (30, 52)	45 (22, 56)	0.868
Male sex, n (%)	56 (58%)	30 (45%)	26 (84%)	**<0.001**
BSA, (m^2)	1.9 (1.7, 2.0)	1.8 (1.7, 2.0)	2.0 (1.8, 2.2)	**0.014**
**Type of pathogenic variant, 66 (68%)**				
PKP2, n (%)	39 (40%)	39 (59%)	0 (0%)	-
DSP, n (%)	21 (22%)	21 (32%)	0 (0%)	-
DSG2, n (%)	3 (3%)	3 (5%)	0 (0%)	-
DSC2, n (%)	3 (3%)	3 (5%)	0 (0%)	-
**Ethnicity**				
White, n (%)	61 (63%)	48 (73%)	13 (42%)	
Asian, n (%)	11 (11%)	4 (6%)	7 (23%)	
Black, n (%)	4 (4%)	3 (5%)	1 (3%)	
Non-specified/mixed, n (%)	21 (22%)	11 (17%)	10 (32%)	**0.017**
**Symptoms**				
Palpitation, n (%)	38 (39%)	26 (39%)	12 (39%)	1.000
Syncope, n (%)	22 (23%)	12 (18%)	10 (32%)	0.192
**Medication**				
Statins, n (%)	3 (3%)	0 (0%)	3 (10%)	**0.030**
Anticoagulant, n (%)	6 (6%)	3 (5%)	3 (10%)	0.380
Antiarrhythmic drugs, n (%)	15 (15%)	9 (14%)	6 (19%)	0.550
Beta blocker, n (%)	22 (23%)	16 (24%)	6 (19%)	0.795

Abbreviations: BSA: body surface area. Data are reported as median (IQR), with p-values from Mann-Whitney U tests; or as n: number (%), with p-values from Fisher's exact tests.

Table S7. Structural progression over time between patients with *PKP2* and *DSP* variants

Markers	Gradient per year measured over 4 years				Interaction p-value
	**Patients with *PKP2* variant (n = 39)**		**Patients with *DSP* variant (n = 21)**		
	Statistics (95% CI)	p-value	Statistics (95% CI)	p-value	
RVOT-PLAX (cm)	0.03 (0.005, 0.05)	**0.020**	0.04 (-0.003, 0.08)	0.067	0.765
RVOT-PSAX (cm)	0.03 (0.001, 0.06)	**0.043**	0.01 (-0.04, 0.06)	0.704	0.474
RV-FAC (%)	-0.4 (-0.9, 0.1)	0.122	-0.2 (-1.4, 0.9)	0.697	0.782
LV-EF (%)	-0.2 (-0.6, 0.2)	0.290	0.2 (-0.6, 1)	0.684	0.423

Abbreviations: RVOT PLAX: right ventricular outflow tract parasternal long axis; RVOT PSAX: right ventricular outflow tract parasternal short axis; LV EF: left ventricular ejection fraction. Longitudinal trends were analysed using a generalized estimating equation approach.

Table S8. Baseline imaging characteristics between patients with *PKP2* and *DSP* variants

**Imaging characteristics**	**Patients with *PKP2* variant (n =39)**	**Patients with *DSP* variant (n=21)**	**p value**
**Echo data**			
RVOT-PLAX (cm)	2.9 (2.4 - 3.4)	2.7 (2.5 - 3.0)	0.340
RVOT-PSAX (cm)	3.0 (2.6 - 3.6)	3.0 (2.9 - 3.3)	0.726
RVEDA (cm2)	22 ± 7	18 ± 6	0.063
RV FAC (%)	44 (35-48)	48 (42-50)	0.129
LVEDV (ml)	89 ± 28	99 ± 36	0.309
LV EF (%)	65 (59-67)	59 (55-66)	0.075
[a] **CMR data**			
RVEDV (ml)	155 (137-197)	183 (120-200)	0.568
RV EF (%)	48 ± 13	46 ± 16	0.779
LVEDV (ml)	142 ± 38	189 ± 44	**0.014**
LV EF (%)	65 ± 10	53 ± 15	**0.041**
[b] LGE present, n (%)	10 (37%)	6 (60%)	0.274

RV and LV LGE, n (%)	1 (4%)	5 (50%)	**0.003**
LV - specific LGE, n (%)	2 (7%)	1 (10%)	1.000
RV - specific LGE, n (%)	7 (26%)	0 (0%)	0.155

Abbreviations: RVOT PLAX: right ventricular outflow tract parasternal long axis; RVOT PSAX: right ventricular outflow tract parasternal short axis; RVEDA: right ventricular end diastolic area; RVFAC: right ventricular fractional area change; LVEDD: left ventricular end-diastolic volume; LVEF: left ventricular ejection fraction; LGE: late gadolinium enhancement. Data are reported as mean±SD, with p-values from independent samples t-tests; median (IQR), with p-values from Mann-Whitney U tests; or as n: number (%), with p values from Fisher's exact tests. a CMR was performed in 28 patients (28 PKP2 carriers and 10 DSP carriers,). b LGE was assessed in 37 patients (27 PKP2 carriers and 10 DSP carriers).

Table S9. Definite ARVC patients (n=15) with Epsilon wave classified as minor criterion per "2020 Criteria"

Patients	Criteria	Diagnosis
Patient 1	-1 major imaging<br>-1 minor depolarisation, (epsilon wave)<br>-1 minor depolarisation<br>- 1 major arrhythmias<br>- 1 major family history	Definite
Patient 2	-1 major imaging<br>-1 minor depolarisation, (epsilon wave)<br>-1 major repolarisation<br>-1 minor repolarisation<br>-1 major arrhythmias	Definite
Patient 3	-1 minor imaging<br>-1 minor depolarisation, (epsilon wave)<br>-1 minor repolarisation	Borderline
Patient 4	-1 major imaging<br>-1 minor depolarisation, (epsilon wave)<br>-1 minor depolarisation<br>-1 major repolarisation<br>-2 minor arrhythmias<br>-1 major family history	Definite
Patient 5	-1 major imaging<br>-1 minor depolarisation, (epsilon wave)<br>-1 minor depolarisation<br>-1 major repolarisation<br>1 minor arrhythmias	Definite
Patient 6	-1 major imaging<br>-1 minor depolarisation, (epsilon wave)<br>-1 major repolarisation<br>-1 minor arrhythmias<br>-1 major family history	Definite
Patient 7	-1 major imaging<br>-1 minor depolarisation, (epsilon wave)<br>-1 minor depolarisation	Definite

	-1 major repolarisation<br>-1 minor repolarisation<br>-1 major arrhythmias<br>-1 major family history	
Patient 8	-1 major imaging<br>-1 minor depolarisation, (epsilon wave)<br>-1 major repolarisation<br>-1 major arrhythmias	Definite
Patient 9	-1 major imaging<br>-1 minor depolarisation, (epsilon wave)<br>-1 minor depolarisation<br>-1 minor repolarisation<br>-1 major arrhythmias<br>-1 major family history	Definite
Patient 10	-1 minor depolarisation, (epsilon wave)<br>-1 major repolarisation<br>-1 major arrhythmias<br>-1 major family history	Definite
Patient 11	-1 major imaging<br>-1 minor depolarisation, (epsilon wave)<br>-1 minor repolarisation<br>-1 major arrhythmias<br>-1 major family history	Definite
Patient 12	-1 major imaging<br>-1 minor depolarisation, (epsilon wave)<br>-1 minor depolarisation<br>-1 minor repolarisation<br>-1 minor arrhythmias<br>-1 minor family history	Definite
[a] Patient 13	-1 major imaging<br>-1 minor depolarisation, (late potential)<br>-1 major repolarisation<br>-1 major family history	Definite
[a] Patient 14	-1 major imaging<br>-1 minor depolarisation, (late potential)<br>-1 major repolarisation	Definite
[a] Patient 15	-1 minor imaging<br>-1 minor repolarisation<br>-1 minor arrhythmias<br>-1 major family history	Definite

[a] *Patients developed Epsilon wave during follow up.*

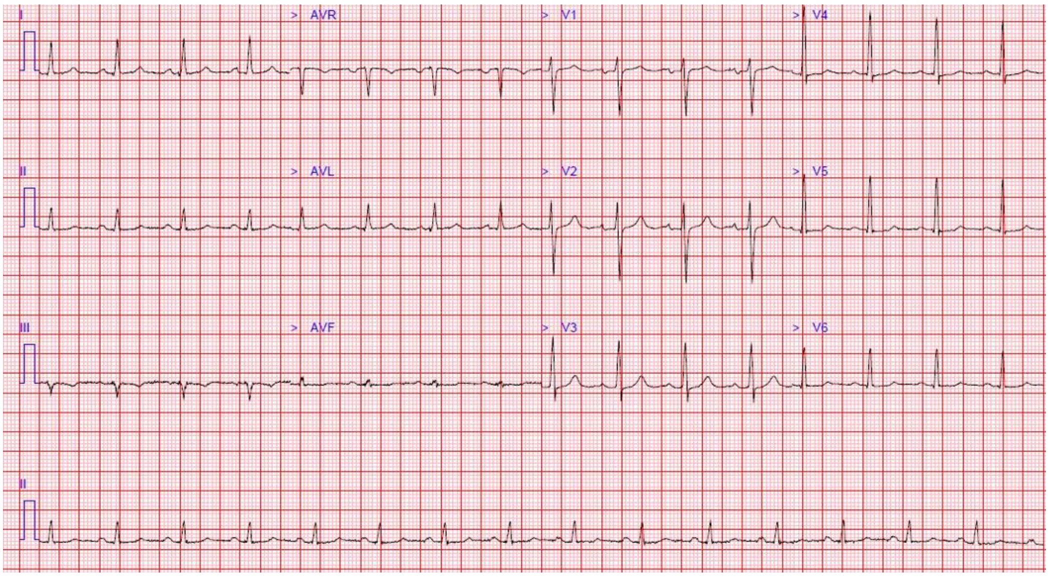

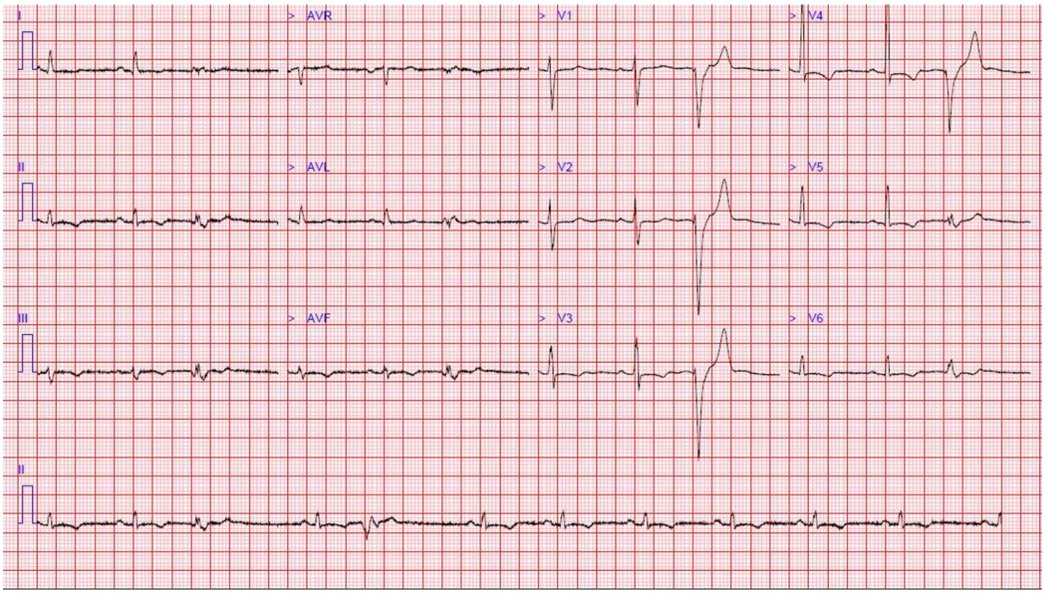

Figure S1. Original baseline and follow up ECG of a pathogenic variant carrier. A 35 year-old female with a *DSP* variant, presented with normal 12 lead ECG (top). During follow-up the patient gained minor repolarisation criteria (T-wave inversion in leads V4-V6, bottom). The patient was known to have premature ventricular contractions (PVC) during the initial 24 Holter monitoring already. PVCs were seen on the follow up ECG (bottom) and a high number of PVCs were seen on the follow up Holter as well.
